# Report of a New Case of Pentasomy X Revealed by Status Epilepticus

**DOI:** 10.7759/cureus.16062

**Published:** 2021-06-30

**Authors:** Nabila Chekhlabi, Amal Haoudar, Hasna Hamdaoui, Nezha Dini

**Affiliations:** 1 Pediatrics, Cheikh Khalifa International University Hospital, Mohammed VI University of Health Sciences, Casablanca, MAR; 2 Anesthesia and Critical Care, Cheikh Khalifa International University Hospital, Mohammed VI University of Health Sciences, Casablanca, MAR; 3 Genetics, Mohammed VI University of Health Sciences, Casablanca, MAR; 4 Pediatrics, The International University Hospital Cheikh Khalifa Ibn Zaid, Casablanca, MAR; 5 Faculty of Medicine and Pharmacy, Mohamed V University, Rabat, MAR

**Keywords:** pentasomy x, 49 xxxxx, children, epilepsy, karyotype

## Abstract

This report describes an exceptional case of X (49, XXXXX) pentasomy in a girl aged three years and five months. She was admitted for recurrent seizures revealing epilepsy. She has growth failure and psychomotor retardation with a deformed face. The malformative assessment did not show any malformation apart from cerebral leukodystrophy. Pentasomy X is a very rare abnormality of the sex chromosomes. It only affects females, in whom three additional X chromosomes are added to the two X normally present. The pathogenesis of pentasomy X is not exactly clear, but it is probably caused by successive maternal nondisjunctions. Epilepsy and cerebral leukodystrophy are a new mode of revelation of this syndrome, never described in the literature.

## Introduction

Pentasomy X (49, XXXXX) is a very rare aneuploidy involving sex chromosome X. It is characterized by a variable phenotype in females. Pentasomy X was first described in 1963, by Kesaree and Wooley, in a two-year-old girl, then only around 30 children with a 49, XXXXX karyotype were reported [1].

The aim of this publication is to describe an additional case with a chromosomal composition 49, XXXXX and to report a new mode of revelation by a state of epilepsy associated with cerebral leukodystrophy.

Comparison of the clinical characteristics of this child with the previously reported cases suggests that syndrome 49, XXXXX is systematically associated with somatic and psychomotor growth retardation as well as varying degrees of malformations.

## Case presentation

The patient was a three-year and five-month-old girl, the last of five siblings, from healthy and unrelated parents (38-year-old mother and 46-year-old father). She was born vaginally at term without perinatal distress. Her birth weight was 2000 g (severe hypotrophy [<3rd percentile]), and her height and head circumference were unknown. The family history was unremarkable and her siblings are healthy and normally neurodeveloped. The infant was admitted to Cheikh Khalifa hospital for febrile status epilepticus with a history of psychomotor delay. She walked at the age of 3 and she is still not speaking. There was no history of an increased incidence of recurrent infections.

**Figure 1:**
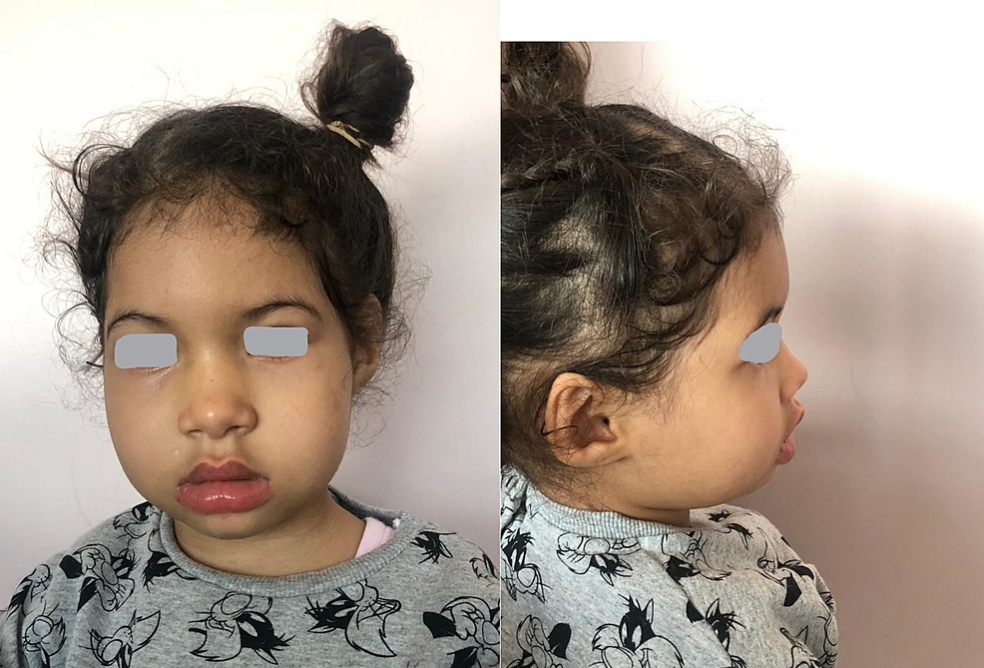
Physical appearance of the patient's face

Her physical examination revealed that she had a body temperature of 40°C, was hemodynamically stable but still had generalized tonicoclonic seizures. Her capillary glycemia was 0.88. Her current weight was 14 kg (25-50th percentile); her length was 86 cm (25th percentile). She also had hypertelorism and oblique eye fissures, upward ears, and normal external genitalia (Figure 1).

She was admitted directly to the intensive care unit where she received medical conditioning, an intravenous infusion of phenobarbital and paracetamol.

**Figure 2:**
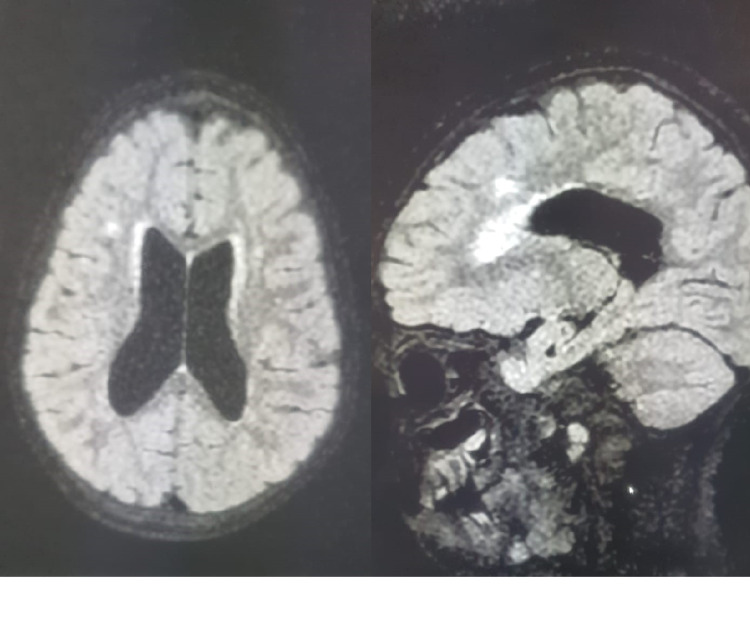
Brain MRI showing moderate leukodystrophy.

After the convulsions had stopped, an infection assessment was made. The lumbar puncture was normal. Laboratory assessment revealed normal c-reactive protein and white blood cell count. There were no metabolic disturbances (natremia, calcemia, lactatemia, and ammoniaemia) and no signs of urinary tract infection. Brain MRI showed cerebral leukodystrophy and electroencephalogram revealed diffuse epileptic abnormalities (Figure 2).

**Figure 3:**
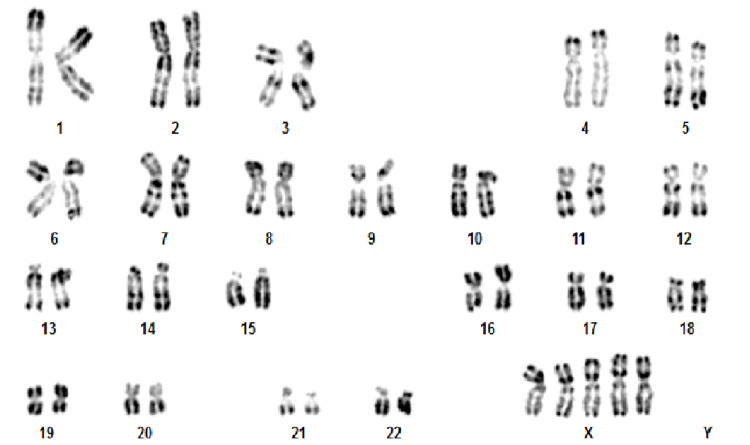
R-band karyotype of the patient demonstrating 49, XXXXX.

No structural eye abnormalities were found at examination by an ophthalmologist. Transthoracic echocardiography and ultrasonography of the urinary tract did not reveal any abnormalities. Auditory evoked potentials did not reveal deafness. The immunoglobulin level was normal. Standard chromosome analysis with R-banding was performed on cultured peripheral blood lymphocytes from the patient and it revealed a 49, XXXXX karyotype in all cells without evidence of mosaicism (Figure 3).

The patient was discharged after five days of hospitalization, under a sodium valproate-based treatment with complete regression of the seizures.

## Discussion

Pentasomy X, also called penta-X syndrome, chromosome X pentasomy, Poly-X, and 49, XXXXX syndrome, is a rare chromosomal abnormality probably due to a nondisjunction during the meiosis [2].

The incidence of pentasomy X is unknown due to the extreme rarity of this aneuploidy. It is much rarer than the 49, XXXXY whose incidence in men is estimated by some authors at 1/85,000 [3,4]. To our knowledge, since 1983, only about 30 isolated cases of X pentasomy have been reported in the literature.

The pathogenesis of pentasomy X is not fully understood: This aneuploid state must result from a dysfunction in meiosis, either maternal or combined maternal and paternal in origin. The X chromosomes of this pentasomy are secondary either to a meiotic nondisjunction, where the two X chromosomes do not separate during the first or second division of gametogenesis, or to a mitotic nondisjunction in the zygote during its development. If the X chromosomes do not separate properly and go on to the next cell division and do not divide fully yet, when the sperm fertilizes the egg, the fetus is left with four X chromosomes from one parent and a fifth X from the other parent (49, XXXXX) [5]. Since in the majority of cases the parents do not carry chromosomal abnormalities, the risk of having another daughter with an X pentasomy is not higher than in the general population.

The features described in karyotype 49, XXXXX is more severe than in X trisomies and tetrasomies [6], including severe mental retardation with delayed language development, significant height delay, facial dysmorphisms, bone abnormalities, and congenital heart defects [7]. In the majority of published postnatal X-pentasomy cases, mental retardation and failure to thrive have been described as constant telltale signs [7]. A moderate deficit in psychomotor development is the most described neurological manifestation. A suspected case of spinal muscular atrophy has been reported in Turkey in an infant with pentasomy X, but confirmatory genetic testing could not be performed [5]. Epilepsy and cerebral leukodystrophy, discovered in our patient, have not been described in the majority of the 49, XXXXX forms published in the literature. The other origins of leukodystrophy are unlikely, since the parents are not consanguineous and the four siblings had normal neurological development. In addition, our patient had no history of perinatal asphyxia, and her metabolic analyses (lactatemia, ammoniaemia, and thyroid workup) were normal. We mention this association (pentasomy X and epilepsy) without being able to confirm a causal link, but we are counting on future publications to report other similar cases.

Common features found in patients previously described in the literature and in our patient were failure to thrive, psychomotor delay, hypertelorism, flat broad nose, mongoloid tilt of palpebral fissures, and normal external genitalia. In contrast, features frequently found in previous patients and not in our patient included a short neck, fifth finger clinodactyly, heart defect, chorioretinal disease, immunoglobulin abnormalities, and increased susceptibility to infections [8]. Despite the normal appearance of the external genitalia, gonadal dysfunction frequently exists in patients with penta-X syndrome [8]. Our patient being three and a half years old, her sexual development remains to be evaluated as well as her bone maturation and the possibility of osteo-articular deformity during growth.

## Conclusions

X-pentasomy is an extremely rare chromosomal abnormality and parental karyotypes are often normal. The most likely cause is the nondisjunction of the maternal X chromosomes during the successive division of two meiosis. This syndrome is frequently characterized by a significant delay in height and deficits in the psychomotor and cognitive domains.

Our case will enrich the literature with epilepsy as an unusual and unreported mode of revelation in the previous cases of 49, XXXXX.
